# MicroRNA-155, induced by FOXP3 through transcriptional repression of *BRCA1*, is associated with tumor initiation in human breast cancer

**DOI:** 10.18632/oncotarget.17816

**Published:** 2017-05-11

**Authors:** Song Gao, Yicun Wang, Meng Wang, Zhi Li, Zhiying Zhao, Raymond X. Wang, Rong Wu, Zhengwei Yuan, Ranji Cui, Kai Jiao, Lizhong Wang, Ling Ouyang, Runhua Liu

**Affiliations:** ^1^ The Second Department of Clinical Oncology, Shengjing Hospital of China Medical University, Shenyang, China; ^2^ Department of Genetics, University of Alabama at Birmingham, Birmingham, Alabama, USA; ^3^ Provincial Key Laboratory on Molecular and Chemical Genetic, Second Hospital of Jilin University, Changchun, China; ^4^ Department of Oncology, Cancer Hospital of Harbin Medical University, Harbin, China; ^5^ Department of General Surgery, Henan Cancer Hospital, Zhengzhou, China; ^6^ School of Computer Science and Engineering, Northeastern University, Shenyang, China; ^7^ Comprehensive Cancer Center, University of Alabama at Birmingham, Birmingham, Alabama, USA; ^8^ Department of Gynecology and Obstetrics, Shengjing Hospital of China Medical University, Shenyang, China

**Keywords:** microRNA, breast cancer, plasma, FOXP3, BRCA1

## Abstract

MicroRNA (miR)-155 is upregulated in breast cancer cells and in sera of patients with breast cancer, but its clinical relevance remains uncertain. The objective of the present effort was to address the transcriptional regulation of miR-155. A bioinformatics analysis of public datasets validated upregulation of miR-155 in tumor cells of patients with breast cancer, particularly those who were at early stages and had triple-negative cancers. The expression profiling and clinical relevance of miR-155 in tumor cells and blood cells were characterized by TaqMan miR assays and, in plasma and exosomes, by nest-quantitative PCR analysis. There was a positive correlation between expression of *FOXP3* and miR-155 in breast cancer cell lines and primary breast cancers. In breast cancer cells, FOXP3 induced miR-155 through transcriptional repression of *BRCA1*. Furthermore, in an Alabama cohort, blood and plasma samples were collected from 259 participants, including patients with breast cancer or benign breast tumors, members of breast cancer families, and matched healthy female controls. For patients with early stage or localized breast cancer, there were high levels of miR-155 in both plasma and blood cells. In cultured breast cancer cells, expression of miR-155 was induced by FOXP3 but was not significantly changed in culture medium or exosomes, suggesting that circulating miR-155 originated from blood cells. These findings reveal a transcriptional axis of FOXP3-BRCA1-miR-155 in breast cancer cells and show that plasma miR-155 may serve as a non-invasive biomarker for detection of early stage breast cancer.

## INTRODUCTION

MicroRNAs (miRs) are small, noncoding RNAs that control expression of target genes through inhibiting protein translation or inducing degradation of mRNA transcripts of target genes [1]. Some miRs, acting as oncogenes (oncomiRs) or tumor suppressor genes, regulate tumor initiation and progression [2, 3]. In lymphoma, miR-155 acts as an oncomiR, and, in breast cancer, it is frequently dysregulated [4]. In breast cancer cells, it has functions in proliferation, apoptosis, angiogenesis, and the epithelial–mesenchymal transition (EMT). Ectopic overexpression of miR-155 enhances proliferation in human breast cancer cells and tumor growth in breast cancer xenografts, while antisense targeting of miR-155 inhibits proliferation, angiogenesis, migration, and invasion, as well as the EMT, but induces cell cycle arrest, apoptosis, and enhances their response to radiation and chemotherapy [5–10]. However, in a mouse 4T1 xenograft model of breast cancer, ectopic expression of miR-155 prevents tumor dissemination from mammary fat pads, but, in lung metastases, it maintains the epithelial phenotype of tumor cells and promotes tumor formation [11]. Thus, there is an inconsistency in these observations between human and mouse breast cancer cells. Since, between mice and humans, one nucleotide of miR-155 (hsa-miR-155-5p: uuaaugcuaaucgugauaggggu; mmu-miR-155-5p: uuaaugcuaauugugauaggggu) is different, miR-155 may have a species-specific effect during tumor progression. In addition, to understand the biological function of miR-155, recent studies have involved screening of a series of miR-155 targets, including *CEBPB* [12], *ERBB2* [13], *FOXO3* [14, 15], *HIF1A* [16, 17], *RAD51* [18], *RHOA* [5], *SATB1* [19], *SOCS1* [6], *TCF4* [11], *TERF1* [20], *VHL* [10], and *ZNF652* [21] in human cancers. In breast cancer cells, there are two negative transcriptional regulators of miR-155, BRCA1 [7] and p63 [21]. However, the mechanism for transcriptional regulation of miR-155 remains largely unknown. Particularly needed is identification of positive transcriptional regulators of miR-155 for identifying new therapeutic targets in human breast cancer.

Clinical investigations reveal that expression of miR-155 is upregulated in human breast cancers [8, 22–25]. Of note, miR-155 is more frequently overexpressed in invasive and metastatic breast tumors than in non-invasive breast tumors [5, 8]. Upregulation of miR-155 in tumor cells is also associated with triple-negative and basal-like breast cancers [10, 24]. However, the clinical significance of miR-155 expression in tumor cells remains uncertain. For example, high levels of miR-155 in tumor cells correlate with either a good prognosis [14] or a poor prognosis [10]. Furthermore, serum miR-155 could serve as a non-invasive biomarker in diagnosis, prognosis, and therapy of breast cancer, for levels of serum miR-155 are higher in patients with breast cancer than in healthy female controls [26–28], suggesting a breast cancer-specific effect on circulating miR-155. However, for patients with breast cancer, data are inconsistent in regard to high levels of serum miR-155, which are associated with either higher [27] or lower [29] tumor stages. Conversely, low levels of serum miR-155 are associated with distant metastasis [26, 30]. Thus, the relationship between circulating miR-155 and tumor progression remains controversial. Circulating miR-155 is decreased in mice and patients after surgery, radiotherapy, or chemotherapy [27, 31]. In addition, most studies have focused on expression of miR-155 in tumor cells and circulation, but, for patients with breast cancer, there is limited evidence of the cell origin of circulating miR-155. In the present study, we investigated a) the clinical relevance of miR-155 in both tumor cells and in circulation during tumor initiation and progression, b) the transcriptional regulation of miR-155, and c) the origin of circulating miR-155 in patients with breast cancer.

## RESULTS

### Characterization of miR-155 expression profiling in human primary breast cancers

**Figure 1:**
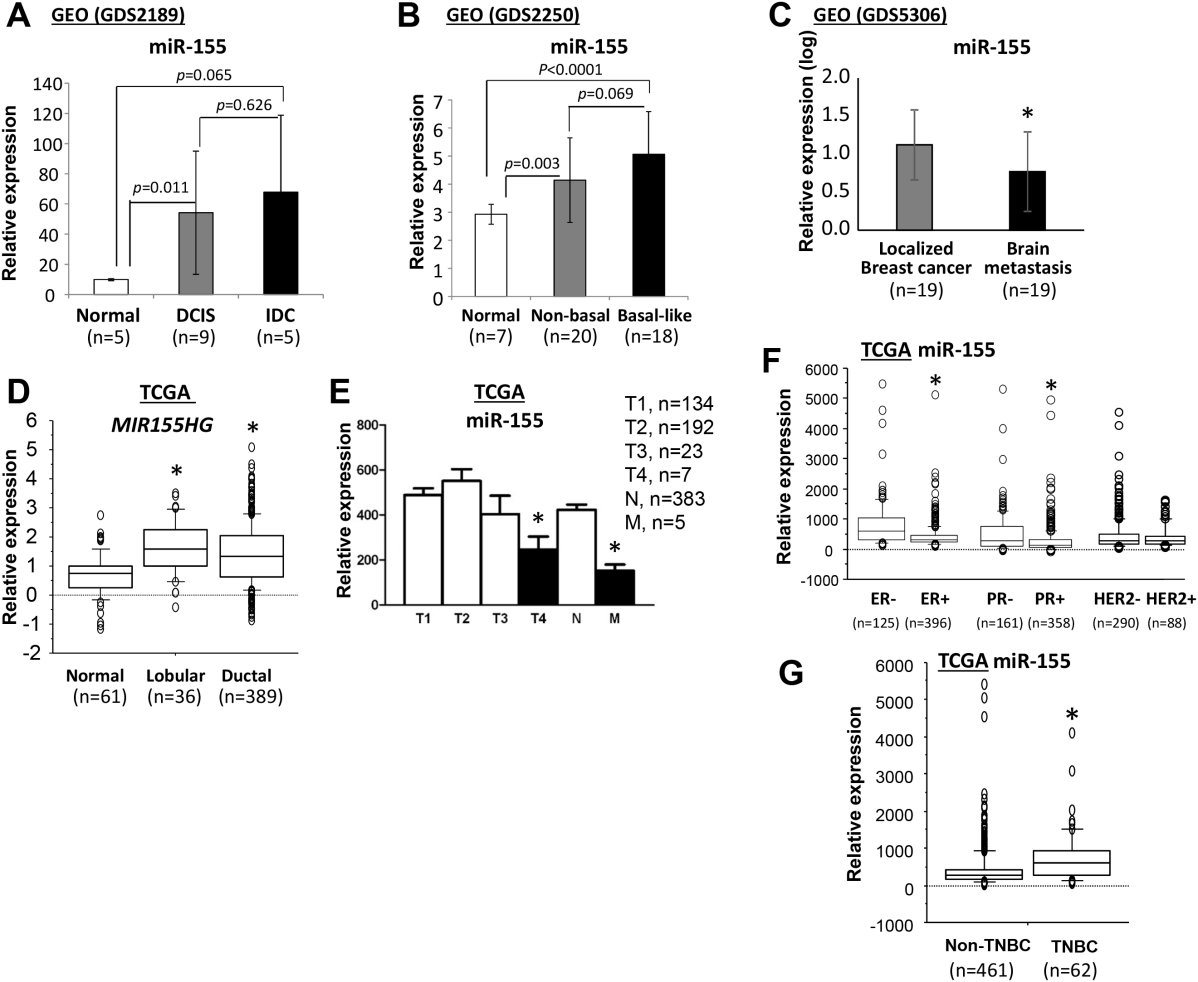
Expression of miR-155 in human normal breast and breast cancer samples **(A-C)** Data analyses were performed using the datasets from NCBI GEO database. Quantification of miR-155 expression **(A)** in samples of normal breast and DCIS and invasive ductal carcinoma, **(B)** in normal breast and non-basal and basal-like breast cancer samples, and **(C)** in localized and brain metastatic breast cancer samples. Data analyses were performed using datasets from the NCI TCGA database. Quantification of miR-155 or *MIR155HG* expression was accomplished **(D)** for normal breast and breast lobular carcinoma and ductal carcinoma samples, **(E)** for breast cancer samples with different stages, **(F)** for breast cancer samples with ER/PR/HER2 status, and **(G)** for non-TNBC and TNBC samples. All data are presented as the means and SD or as the medians and interquartile ranges. *p<0.05, using a two-tailed *t* test in samples with normal distributions or using a Mann-Whitney test for samples with non-normal distributions. DCIS, ductal carcinoma *in situ*; IDC, invasive ductal carcinoma; NCBI, National Center for Biotechnology Information; GEO, Gene Expression Omnibus; NCI, National Cancer Institute, TCGA, The Cancer Genome Atlas; ER, estrogen receptor; PR, progesterone receptor; HER2, human epidermal growth factor receptor 2; TNBC, triple negative breast cancer; SD, standard deviation.

To assess the clinical relevance of miR-155 in human primary breast cancers, the expression profile of miR-155 was characterized by bioinformatics analysis of public datasets from a) the National Center for Biotechnology Information (NCBI) Gene Expression Omnibus (GEO) and b) the National Cancer Institute (NCI) The Cancer Genome Atlas (TCGA). As determined by analysis of the NCBI GEO data, expression of miR-155 was more than 5-fold higher in ductal carcinoma *in situ* (DCIS) and intraductal (IDC) tumors than in normal breast tissues (Figure 1A). Of note, expression of miR-155 was higher in basal-like breast tumors relative to non-basal like breast tumors, but this increase was not statistically significant (*p*=0.069, Figure 1B). However, expression of miR-155 was lower in tumors with brain metastases than in localized breast tumors (Figure 1C). As determined by analysis of the NCI TCGA data, expression of *MIR155HG*, a host gene of miR-155, was approximately 2-fold higher in breast tumors than in normal breast controls, but there was no significant difference between lobular and ductal breast tumors (Figure 1D). Since the mature form of miR-155 is encoded by exon 3 of the non-coding RNA, *MIR155HG* [32], expression levels of miR-155 should be consistent with that of its host gene, *MIR155HG*. Expression of miR-155 was also higher in T1-3 breast tumors than in T4 breast tumors or metastatic breast tumors (Figure 1E). Likewise, there was high expression of miR-155 in estrogen receptor (ER)- and progesterone receptor (PR)-negative breast cancers and in triple-negative breast cancers (TNBCs), but there was no difference associated with human epidermal growth factor receptor 2 (HER2) status (Figure 1F–1G).

### FOXP3 induces miR-155 expression in human breast cancer cells

**Figure 2:**
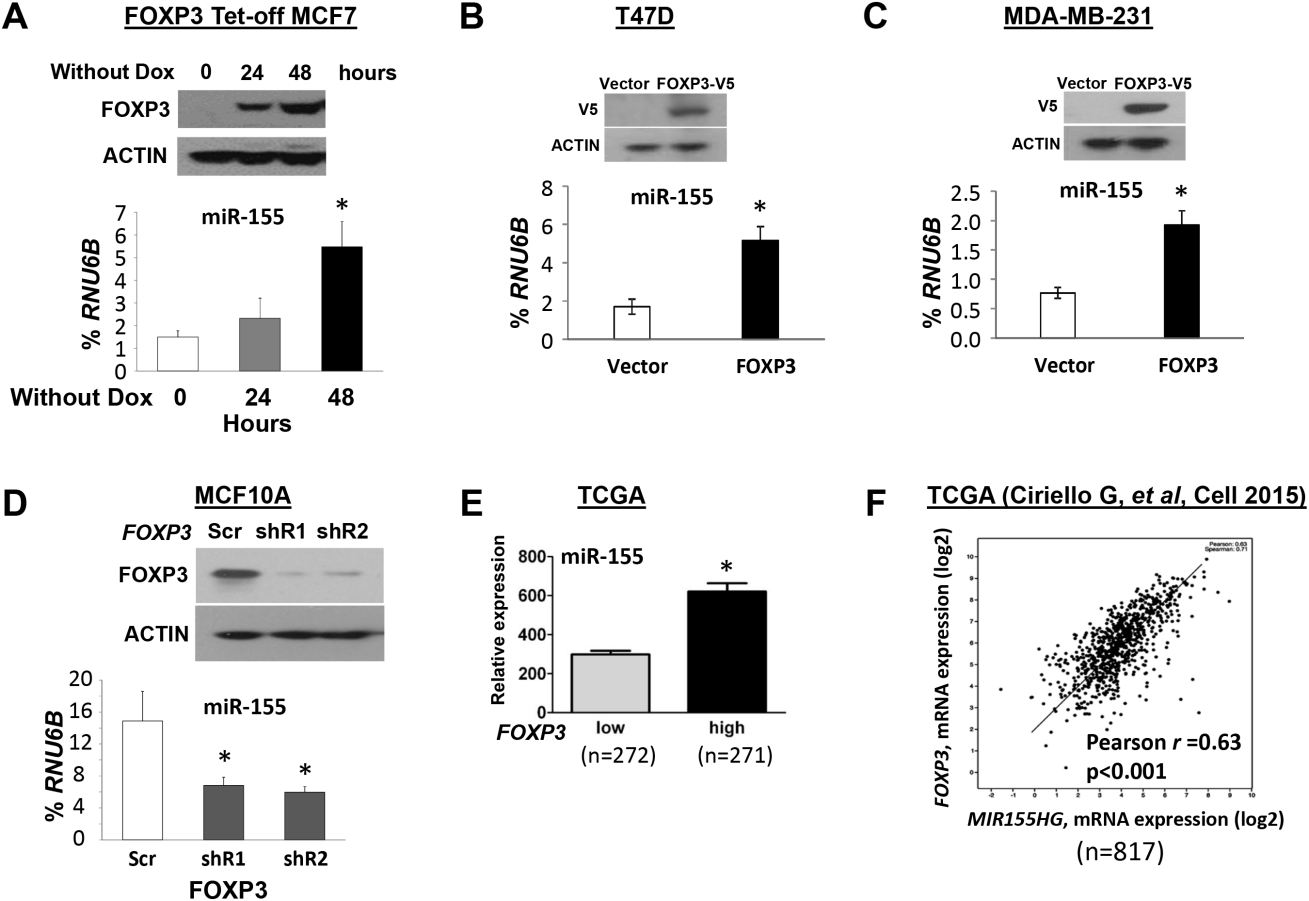
FOXP3 induces miR-155 expression in human breast cancer cells **(A)** Expression of miR-155 in FOXP3 Tet-off MCF7 cells. Top panels: Representative Western blots showing FOXP3 expression at 0, 24, and 48 hours in cells without doxycycline (Dox). Bottom graphs: Quantification of miR-155 expression as a percentage of endogenous control *RNU6B* expression at 0, 24 and 48 hours. **(B)** The expression levels of miR-155 in MCF10A cells. Top panels: Representative Western blots showing FOXP3 expression after transfection of cells with *FOXP3* shRNA or scramble control. Bottom graphs: Quantification of miR-155 expression after transfection as a percentage of *RNU6B* expression. **(C)** and **(D)** The expression of miR-155 in T47D and MDA-MB-231 cells. Top panels: Representative Western blots showing FOXP3 expression after *FOXP3*-V5 and empty vector transfection. Bottom graphs: Quantification of miR-155 expression as a percentage of *RNU6B* expression after transfection. **(E)** Quantification of *FOXP3* and miR-155 expression in human breast cancer samples from a TCGA dataset. The *FOXP3*^low^ and *FOXP3*^high^ subgroups are divided by a median of *FOXP3* expression. **(F)** Pearson's correlation analysis for a relationship between expressions of *FOXP3* and *MIR155HG* in breast cancer samples from a TCGA dataset. All data in (A-D) are presented as the means and SD of triplicates. *p<0.05, using a two-tailed *t* test. In figure (A-D), all experiments were repeated three times.

In contrast to BRCA1 [7] and p63 [21], which are transcriptional repressors of miR-155, FOXP3, a member of the forkhead-box/winged-helix transcription factor family, is a transcriptional inducer of miR-155 in the breast cancer cell lines, BT549 and MDA-MB-231 [19]. Likewise, FOXP3 also induces expression of miR-155 [33], which contributes to the development of regulatory T cells [34]. In our previous studies, we demonstrated that, in breast and prostate cancers, *FOXP3* is an X-linked tumor suppressor gene [35, 36]. To determine if, in breast cancer cells, miR-155 is induced by FOXP3, the expression levels of miR-155 were monitored in FOXP3 Tet-off MCF7 (ER^+^, PR^+^, HER2^-^) cells without doxycycline (Dox) at 0, 24, and 48 hours [37, 38]. As shown in Figure 2A, miR-155 expression was increased in a time-dependent manner after FOXP3 induction. To substantiate this observation, wild-type *FOXP3* was transfected into breast cancer cell lines T47D (ER^+^, PR^+^, HER2^-^) and MDA-MB-231 (ER^-^, PR^-^, HER2^-^), which have low expression of *FOXP3* [36]. After *FOXP3* transfection, the expression of miR-155 was increased 2- to 3-fold (Figure 2B–2C). Furthermore, *FOXP3* was knocked down by shRNAs in an immortalized human epithelial cell line, MCF10A, which normally expresses *FOXP3* [37, 39]. After *FOXP3* silencing, the expression of miR-155 was more than 2-fold reduced (Figure 2D). In addition, in TCGA datasets, tumor samples of patients with breast cancer were divided into two subgroups (*FOXP3^low^* and *FOXP3*^high^) by a medium value of *FOXP3* expression. As shown in Figure 2E, high levels of miR-155 were present in *FOXP3*^high^ tumors relative to those in *FOXP3*^low^ tumors. Likewise, as supported by our analysis of the TCGA dataset in a previous study [40], Pearson's analysis revealed a positive correlation (*r*=0.63, p<0.001) between expressions of *FOXP3* and *MIR155HG* in primary breast cancers (Figure 2F).

### Identification of a FOXP3-BRCA1-miR-155 axis in human breast cancer cells

**Figure 3:**
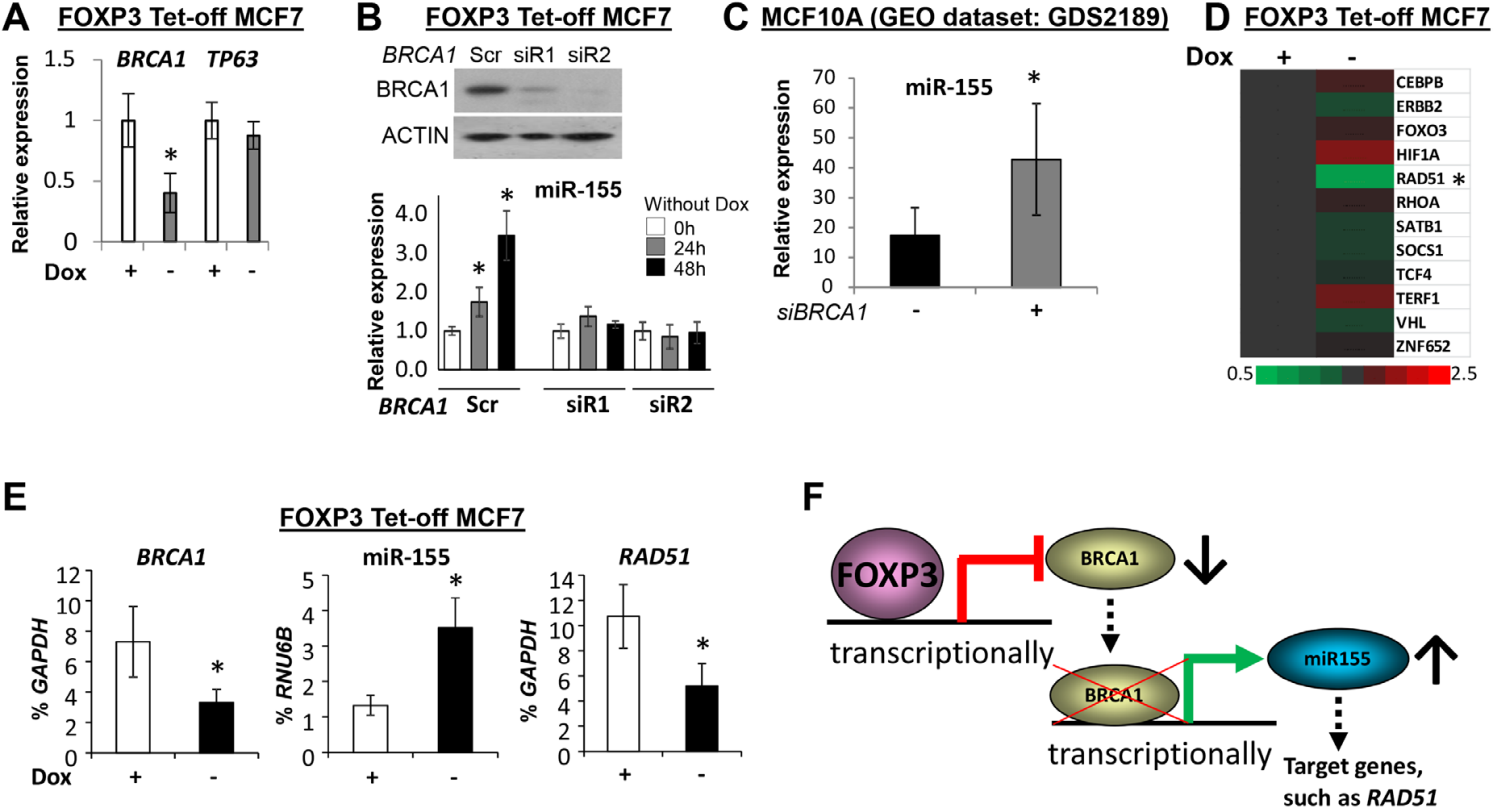
FOXP3-BRCA1-miR-155 axis in human breast cancer cells **(A)** The relative expression levels of *BRCA1* and *TP63* in FOXP3 Tet-off MCF7 cells with or without Dox. The expression of genes in cells with Dox is a reference, 1.0. **(B)** The expression of miR-155 in FOXP3 Tet-off MCF7 cells before and after *BRCA1* silencing. Top panels: Representative Western blots showing BRCA1 expression after transfection with *BRCA1* siRNAs or scramble control in the cells. Bottom graphs: Quantification of miR-155 expression as a percentage of *RNU6B* expression after transfection in the cells. The expression of miR-155 in cells with Dox as a reference, 1.0. Scr, scramble; siR, siRNA. **(C)** Quantification of miR-155 expression after *BRCA1* siRNA silencing in MCF10A cells (data from a NCBI GEO dataset). *siBRCA1*, *BRCA1* siRNA. **(D)** Heat map depiction of alterations in miR-155 target gene expression in FOXP3 Tet-off MCF7 cells was generated from Affymetrix Human U133 plus 2.0 microarrays (EMBL-EBI, accession number E-MTAB-73). **(E)** Expression levels of *BRCA1*, miR-155, and *RAD51* as a percentage of *GAPDH* or *RNU6B* expression in FOXP3 Tet-off MCF7 cells with or without Dox. **(F)** Schematic representation of the FOXP3-BRCA1-miR-155 axis in human breast cancer cells. All data are presented as the means and SD of triplicates. *p<0.05, using a two-tailed *t* test. For Figures A, B, and E, all experiments were repeated three times.

To understand the regulation of miR-155 by FOXP3, the FOXP3 chromatin immunoprecipitation-sequencing data in our previous study were reanalyzed [41]. However, no direct binding signals of FOXP3 were found at loci of miR-155 and its host gene, *MIR155HG* (Supplementary Figure 1). Since FOXP3 is a transcriptional repressor of *BRCA1* [42], a transcriptional repressor of miR-155 [7], *BRCA1* may be a mediator for transcriptional regulation of miR-155 by FOXP3 in human breast cancer cells. A binding signal of FOXP3 in the proximal promoter region of *BRCA1* was also functionally validated [42] (Supplementary Figure 2). After FOXP3 induction in FOXP3 Tet-off MCF7 cells, expression of *BRCA1* was decreased [42], but expression of *TP63* was not changed (Figure 3A). Of note, transfection of *BRCA1* shRNAs into FOXP3 Tet-off MCF7 cells reduced the induction of miR-155 by FOXP3 (Figure 3B), indicating the existence of a transcriptional mediator, *BRCA1*, between FOXP3 and miR-155 in human breast cancer cells. As supported by further analysis of a GEO dataset, miR-155 was increased in MCF10A cells after *BRCA1* silencing (Figure 3C). Furthermore, using our previous microarray data [41], we analyzed the expression profile of miR-155 target genes, including *CEBPB* [12], *ERBB2* [13], *FOXO3* [14, 15], *HIF1A* [16, 17], *RAD51* [18], *RHOA* [5], *SATB1* [19], *SOCS1* [6], *TCF4* [11], *TERF1* [20], *VHL* [10], and *ZNF652* [21], in FOXP3 Tet-off MCF7 cells. *RAD51* was reduced at 48 hours after FOXP3 induction (Figure 3D). The upregulation of miR-155 and downregulation of *BRCA1* and *RAD51* after FOXP3 induction were also validated by TaqMan miR assays and quantitative PCR (qPCR) (Figure 3E). This FOXP3-BRCA1-miR-155 axis was also confirmed in the FOXP3-V5 transfected MDA-MB-231 cells (Supplementary Figure 3). These data indicate the presence, in human breast cancer cells, of a transcriptional axis of FOXP3-BRCA1-miR-155 (Figure 3F).

### High levels of plasma miR-155 are associated with early-stage breast cancer

**Figure 4:**
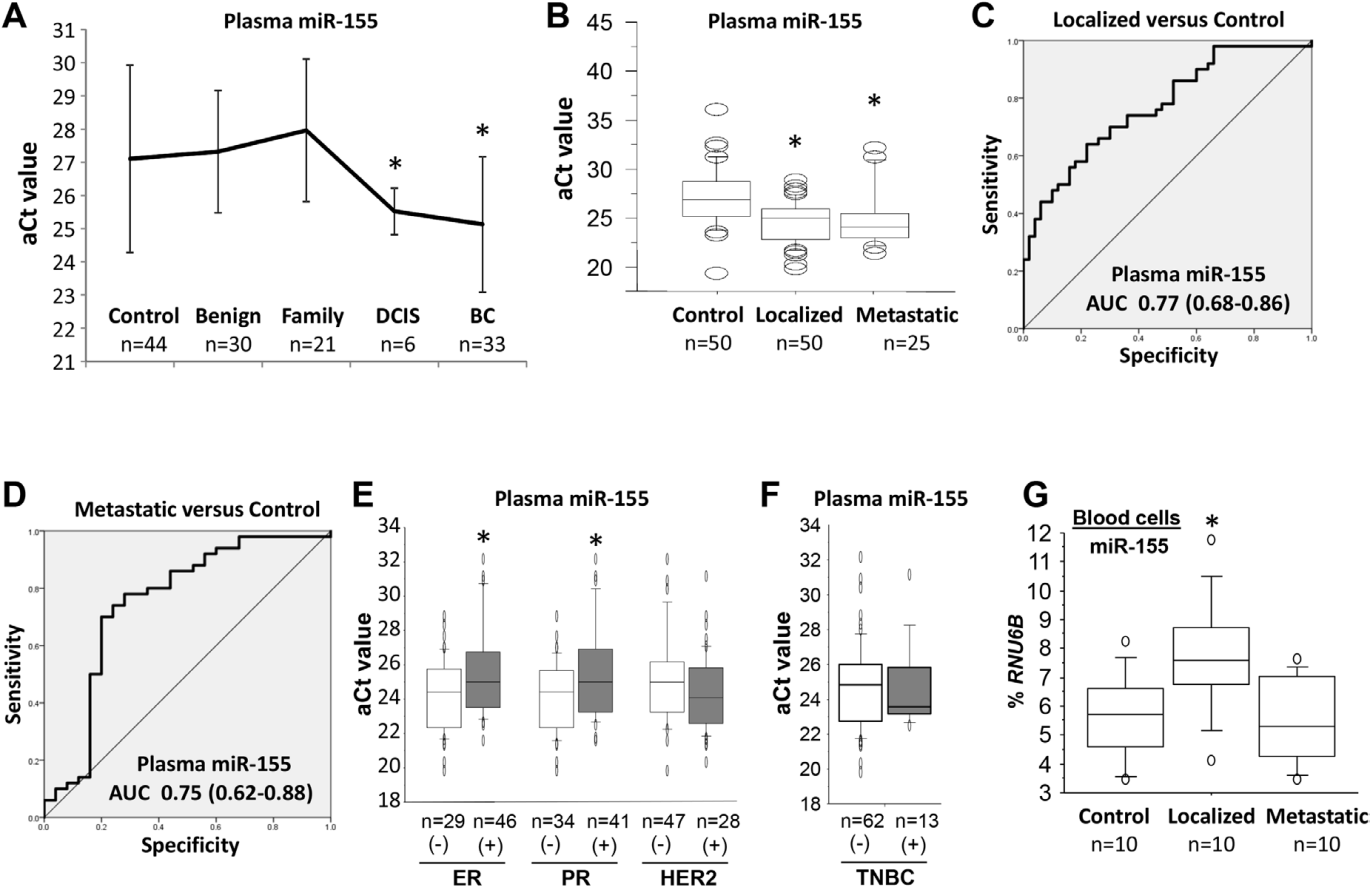
The expression of miR-155 in plasma and blood cells of patients with breast cancer **(A)** The relative expressions of plasma miR-155 in healthy female controls, patients with benign breast tumors, women with a family history of breast cancer, patients with DCIS, and patients with invasive breast cancers. The adjusted Ct (aCt) value of miR-155 was normalized to cel-miR-39. * *p*<0.05 breast cancer group *vs*. control group (one-way ANOVA followed by protected least significant difference test). DCIS, ductal carcinoma *in situ*; BC, breast cancer. **(B)** The relative expression of plasma miR-155 in healthy female controls and patients with localized and metastatic breast cancers. The aCt value for miR-155 was normalized to cel-miR-39. * *p*<0.05 breast cancer group *vs*. control group (two-tailed *t* test). **(C)** and **(D)**, sensitivity and specificity of plasma miR-155 for patients with localized or metastatic breast cancers relative to healthy female controls. Receiver operating characteristic curves are shown for miR-155. AUC, area under the curve. **(E)** and **(F)** The relative expression of plasma miR-155 in breast cancer patients with ER/PR/HER2 status or TNBC.* *p*<0.05 positive group *vs*. negative group (two-tailed *t* test). **(G)** The expression of miR-155 as a percentage of *RNU6B* expression in blood cells of healthy female controls and patients with localized and metastatic breast cancers.* *p*<0.05 breast cancer group *vs*. control group (two-tailed *t* test). All experiments were repeated three times.

To determine the clinical relevance of plasma miR-155, two independent, hospital-based cohorts were obtained from an Alabama population. Demographic and other variables, including age, ethnicity, ER/PR/HER2 status, and clinical factors are summarized in Table 1. In the first cohort, the plasma levels of miR-155 were assessed for 33 patients diagnosed with breast cancer, 6 patients with DCIS, 30 patients with benign breast tumors, 21 women with a family history of breast cancer, and 44 healthy female controls. The plasma levels of miR-155 were higher [a lower adjusted Ct (aCt) value correlated with a higher level] for patients with DCIS or invasive breast cancer than in those of other groups (Figure 4A). There was no significant difference in plasma levels of miR-155 between patients with benign breast tumors, women with a family history of breast cancer, and healthy controls. In the second cohort, all individuals were Caucasian women. The plasma levels of miR-155 were examined for 50 patients with local breast cancer and 25 patients with lymph node or distant metastases. A comparison of data for age- and ethnicity-matched healthy female controls showed that higher levels (lower aCt values) of plasma miR-155 were present for patients with localized breast cancers (6.2-fold, *p*<0.001) and those with metastatic breast cancers (4.7-fold, *p*=0.004) (Figure 4B). However, there was no significant difference between patients with localized and metastatic breast cancers (Figure 4B). Furthermore, although levels of plasma miR-155 were higher for patients with lymph node metastases than for healthy female controls or patients with localized breast cancer, no significant difference was found between healthy female controls and patients with distant metastasis (Supplementary Figure 4). To assess the sensitivity and specificity of plasma miR-155 for its diagnostic potential, a receiver operating characteristic (ROC) analysis was accomplished to determine the capacity of plasma miR-155 to differentiate patients with localized tumors (Figure 4C) or metastatic tumors (Figure 4D) from healthy female controls by use of area-under-the-curve (AUC) values. AUC values for plasma miR-155 were 0.77 (95% confidence intervals: 0.68, 0.86) for patients with localized tumors and 0.75 (0.62, 0.88) for patients with metastatic tumors (Figure 4C–4D). In addition to assessment of ER/PR/HER2 status, plasma levels of miR-155 were evaluated for patients with ER- and PR-negative tumors, but no significant differences were found between those with HER2-positive and -negative tumors or between those with and without TNBC tumors (Figure 4E–4F). Likewise, plasma levels of miR-155 were different between patients with various tumor grades (Supplementary Figure 5) and between patients with tumor subtypes, including invasive ductal and lobular carcinomas (Supplementary Figure 6).

**Table 1:** Characteristics of human subjects

Categories	First cohort			Second cohort		
	BC *^a^*	Benign *^b^*	Family *^c^*	Control *^d^*	BC *^a^*	Control *^d^*
Total number	39	30	21	44	75	50
Recruiting time years	2004-2014	2004-2014	2004-2014	2004-2014	2004-2014	2004-2014
Median age (range) years	51 (30-73)	51 (40-63)	47 (33-71)	50 (36-70)	52 (32-75)	52 (37-71)
Race						
Caucasian	26	28	20	36	75	50
African-American	11	1	1	7	0	0
Other	2	1	0	1	0	0
ER status						
Positive	26				46	
Negative	4				29	
Unknown	9				0	
PR status						
Positive	24				41	
Negative	5				34	
Unknown	10				0	
HER2 status						
Positive	6				28	
Negative	25				47	
Unknown	8				0	
Histological subtype						
Ductal	30				58	
Lobular	2				9	
Both	2				0	
Unclassified	5				8	
Tumor grade						
Well	2				4	
Moderate	13				38	
Poor	17				33	
Unknown	7				0	
Tumor stage						
Localized						
DCIS (pTis)	6				0	
Early (pT1-2N0M0)	15				42	
Advanced (pT3-4N0M0)	4				8	
Metastatic						
Regional (pT1-4N1-3M0)	11				15	
Distant (pT1-4M1)	3				10	

*^a^* Breast cancer patients.

*^b^* Benign breast tumor patients.

*^c^* Normal healthy women with family history of breast cancer.

*^b^* Normal healthy women without family history of breast cancer.

### Potential cell origin of plasma miR-155 in patients with breast cancer

**Figure 5:**
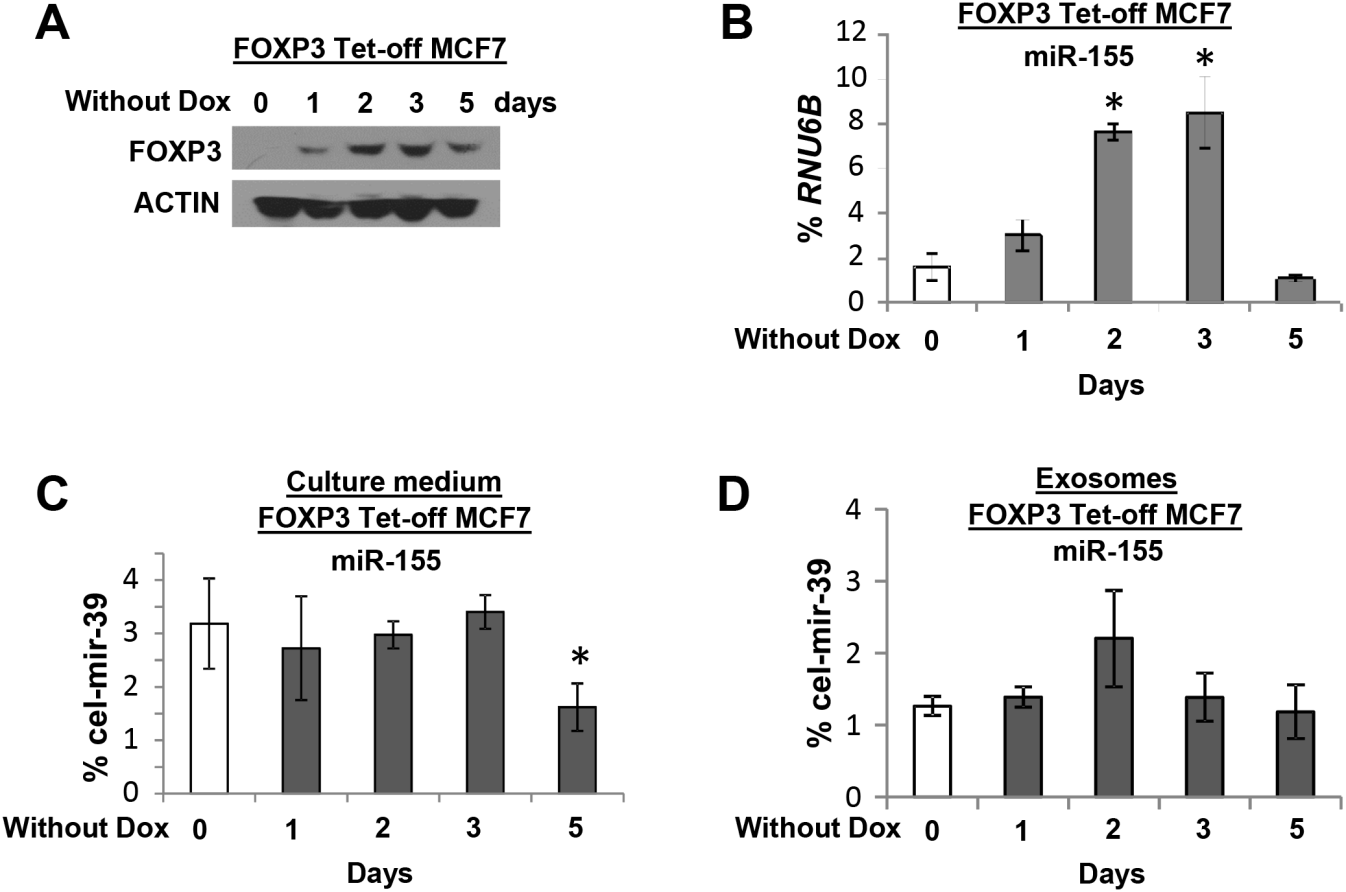
The expression of miR-155 in culture medium and exosomes of FOXP3 Tet-off MCF7 cells **(A)** Representative Western blots showing FOXP3 expression at 0, 1, 2, 3, and 5 days without Dox. The expression levels of miR-155 **(B)** in FOXP3 Tet-off MCF7 cells as a percentage of *RNU6B* expression, **(C)** in culture medium (exosome free) as a percentage of *cel-mir-39* expression, and **(D)** in exosomes as a percentage of *cel-mir-39* expression at 0, 1, 2, 3, and 5 days without Dox. * *p*<0.05 without Dox group *vs*. with Dox group (one-way ANOVA followed by protected least significant difference test). All experiments were repeated three times.

To determine the potential cell origin of plasma miR-155, the levels of miR-155 were measured in peripheral blood cells from 10 patients with local breast tumors, 10 patients with distant metastases, and 10 healthy female controls. The levels of miR-155 were higher for patients with localized tumors than for patients with distant metastases or for healthy female controls (Figure 4G). Next, to characterize the source of plasma miR-155, an analysis of FOXP3 Tet-off MCF7 cells was performed to measure the secretion of miR-155 into the culture medium. Following miR-155 induction by FOXP3 in cultures without Dox at 0 to 5 days (Figure 5A–5B), the extracellular levels of miR-155 were not significantly changed in the cell culture medium (Figure 5C).

Exosomes are nano-sized (50-150 nm) membrane vesicles secreted by most cell types, and tumor cells secrete more than 3-fold more exosomes than normal cells [43]. Exosomes from culture medium were isolated, and their levels of exosomal miR-155 were measured. Although levels of exosomal miR-155 appeared to be increased at day 2, no statistical difference was found (Figure 5D). These data suggest that the plasma miR-155 in patients with breast cancer is derived from blood cells and not from tumor cells.

## DISCUSSION

In breast cancer cells [8, 22–25] and in circulation [26–28], miR-155 is upregulated, but its clinical relevance remains debatable. Although, in tumor cells, high expression levels of miR-155 are reported to be associated with invasive and metastatic breast tumors [5, 8], our analysis of NCI TCGA datasets suggest that low levels of miR-155 are related to invasive (T4) and metastatic (M1) breast tumors. Furthermore, low levels of serum miR-155 are reported to correlate with increased tumor stages [26, 29, 30], but our data indicate that high levels of plasma miR-155 are associated with early stage (DCIS and M0) breast cancer and that there is no difference between localized (M0) and metastatic (N1-3 and M1) breast cancers. In addition, our and other data indicate that miR-155 is highly expressed in ER- and PR-negative tumors and in TNBCs [24, 44]. However, the relationship between circulating miR-155 and ER/PR/HER2 status has remained uncertain [30, 45]. The present data show that high levels of plasma miR-155 are associated with ER- and PR-negative tumors, but not TNBCs.

There are different results for circulating miR-155 levels between previous studies and the present study of patients with breast cancer, which may be caused by various intrinsic and extrinsic factors: 1) methodologies; 2) materials (serum versus plasma, size and type of samples); 3) endogenous controls; and 4) individuals (e.g., age, ethnicity, food and drug consumption). The major differences are likely to be materials and endogenous controls (Supplementary Table 2). Previous studies have involved use of serum to evaluate circulating miR-155 in patients with breast cancer. However, serum is obtained after clotting the whole blood, but miRNA can be released from blood cells during the coagulation process [46]. To avoid thepotentialrelease of miR-155 from blood cells, we selected plasma to estimate the circulating miR-155. In addition, there is a lack of endogenous controls for normalization of circulating miR-155 data. Several small RNA species, such as 18S RNA [28], let7a [29], and miR-16 [26, 30], are used as endogenous controls, but they are either not detectable in circulation or have potential variations across different samples [46]. In the present study, *C. elegans* cel-miR-39 was used as a reference for control optimization [27, 45, 47], and the relative quantity of plasma miR-155 was determined by use of the aCt method with cel-miR-39 [47–49]. For future studies, however, optimal, robust, and reproducible methods for detection of miR-155 in circulation are required.

Recent evidence suggests that most circulating miRs originate from blood cells and endothelial cells [50, 51], but tumor cells and organs, such as liver, lung, and kidney, are also considered to be potential sources of circulating miRs [52], suggesting a complexity of the origin of miRs in circulation [53]. Our analysis shows that miR-155 in culture medium and exosomes appears to be unchanged in FOXP3 Tet-off MCF7 cells through induction of miR-155 by FOXP3. However, for patients with breast cancer, our data reveal that miR-155 is elevated in both plasma and blood cells, especially in patients with localized breast cancer, suggesting that the origin of circulating miR-155 is from blood cells and not from tumor cells. MiR-155 is involved in the homeostasis and function of the immune system and is increased in activated B and T lymphocytes [54–56]. It also acts as an effector of immunosurveillance to inhibit the early stages of breast cancer development [57]. Further, miR-155 knockdown accelerates breast tumor growth by impairing activation of tumor-associated macrophages [57]. Since our data show an increase of miR-155 in both plasma and blood cells of patients with early breast cancer, it would be of interest to determine, in future studies, if an immunologic response during early stages of breast cancer causes the high levels of miR-155 in circulation.

FOXP3 targets and transcriptionally inhibits the breast cancer suppressor gene, *BRCA1*, which reduces the radioresistance of breast cancer cells [42]. In the present study, we identified a FOXP3-BRCA1-miR-155 axis in breast cancer cells. FOXP3 functions as a tumor suppressor in breast cancer cells [36] but also induces expression of oncogenic miR-155 through inhibition of *BRCA1*, representing a heterogeneous network of the target genes affected in FOXP3-mediated tumor suppression. Furthermore, *BRCA1* is involved in development of familial breast cancer. Since BRCA1 epigenetically inhibits miR-155 [7], it is appropriate to test the function of miR-155 in familial breast cancer. In addition, although miR-155 is unlikely to be a direct target of FOXP3 in breast cancer cells, FOXP3 directly targets and induces expression of miR-155 during development of regulatory T cells [33, 34]. Thus, it would be interesting to determine if the FOXP3-BRCA1-miR-155 axis has a similar role in regulatory T cells.

In conclusion, we summarize the expression profiling and clinical characterization of miR-155 in tumor cells, plasma, and blood cells of patients with breast cancer. Upregulation of miR-155 in human breast tumor cells is most likely related to tumor initiation but not to tumor metastasis, and is more favorable to specific tumor subtypes, such as basal-like and TNBCs. Furthermore, miR-155 in tumor cells is associated with transcriptional regulation by FOXP3 through BRCA1, subsequently controlling miR-155 and its targets, such as *RAD51*. Further, plasma miR-155 may be a tool for non-invasive detection of early-stage breast cancer. Although the cell origin of circulating miR-155 remains unclear, our data suggest that it is derived from blood cells in the circulation.

## MATERIALS AND METHODS

### Cell lines, antibodies, and reagents

Breast cancer cell lines, MCF7, T47D, MDA-MB-231, and MCF10A, were obtained from the American Type Culture Collection (Manassas, VA). Cell lines were authenticated by examination of morphology and growth characteristics and were confirmed to be mycoplasma-free. Cells were maintained in Dulbecco's Modified Eagle's medium supplemented with 10% fetal bovine serum (Life Technologies, Grand Island, NY) and cultured for less than 6 months. FOXP3-Tet-off MCF7 cells were established and maintained in Dox (10 μg/ml), as described previously [38, 41, 58]. Specific primary antibodies were used to detect the following proteins: FOXP3 (ab450, 1:2,000, ABCAM, Cambridge, MA) and BRCA1 (#9010, 1:1,000, Cell Signaling, Danvers, MA). The pEF1-FOXP3-V5 vector [35] or empty pEF1 vector was transfected into cells using FuGENE6 (Promega, Madison, WI). *FOXP3* short hairpin RNAs (shRNAs) and *BRCA1* small interfering RNAs (siRNAs) are listed in Supplementary Table 1.

### Human subjects

The present study, involving human subjects, was reviewed and approved by the Institutional Review Board for Human Use at University of Alabama at Birmingham (UAB). Blood and plasma samples were obtained from the Tissue Procurement Shared Facility at the Comprehensive Cancer Center, UAB, with informed consent from all subjects. Between 2004 and 2014, the Tissue Collection and Banking Facility at UAB obtained plasma samples from approximately 1,100 participants with surgically resected breast cancers and controls. For the present study, we selected the plasma from 259 human subjects, including 114 patients with breast cancer, 30 patients with benign breast tumors, 21 women with a family history of breast cancer, and 94 healthy female controls (Table 1). All breast cancer patients were diagnosed by a pathologist for histological confirmation of breast cancer subtype or benign tissues. The pathological stage of breast cancer at the time of diagnosis was determined by use of the Tumor-Node-Metastasis (TNM) system. Tumor grading was based on specimens corresponding to grade 1 (well-differentiated), grade 2 (moderately differentiated), or grade 3 (poorly differentiated).

Human subjects were divided into two independent cohorts for assessment and validation of plasma miR-155 as a potential biomarker. In the first cohort, we obtained plasma from 134 human subjects, including 33 patients with invasive breast cancer (19 cases with pT1-4, 11 cases with N1-3, and 3 cases with M1), 6 patients with non-invasive breast cancer (ductal carcinoma *in situ*, DCIS, pTis), 30 patients with benign breast tumors, 21 women with a family history of breast cancer, and 44 healthy female controls. In the second cohort, plasma was obtained from 125 human subjects in a Caucasian population, including 50 patients with local breast cancer (42 cases with pT1-2N0M0 and 8 cases with pT3-4N0M0) and 25 patients with metastatic breast cancer [15 cases with lymph node only involvement (N1-3) after surgery, and 10 cases with distant metastatic disease (i.e., lungs, liver, bones) (M1) diagnosed after surgery], and 50 healthy female controls. Also, we obtained blood cells from 30 of 125 human subjects, including 10 patients with local breast cancer (pT1-4N0M0) and 10 patients with metastatic breast cancer (distant metastasis), and 10 healthy female controls. The ER/PR/HER2 status in patients with breast cancer was ER (46 positive and 29 negative cases), PR (41 positive and 34 negative cases), and HER2 (28 positive and 47 negative cases). Tumor grades for patients with breast cancer were well-differentiated tumor (4 cases), moderately differentiated tumor (38 cases), and poorly differentiated tumor (33 cases). Tumor types in patients with breast cancer were invasive lobular carcinomas (58 cases), invasive ductal carcinomas (9 cases), and unclassified (8 cases). All healthy female controls were identified by a routine health visit and were matched with patients for age, reproductive status, region of residence, and duration of plasma storage.

### Blood and plasma collection

Blood was collected in EDTA tubes (BD Biosciences), and isolation of plasma and blood cells was accomplished by centrifugation within 4 hours of collection. To avoid the release of miRs from blood cells during the coagulation process [46], miRs in plasma were assessed. Cell-free plasma and blood cells were stored at -80°C until analysis.

### Exosome isolation

Cell culture media was centrifuged at 300 × g for 10 min to clear cells and large debris. The supernatant was centrifuged at 2000 × g for 20 min and then at 10,000 × g for 30 min to remove residual debris. The remaining supernatant was subjected to ultracentrifugation at 100,000 × g for 70-120 min to pellet the exosomes [59], which were suspended in PBS for further analysis. The miRs from exosomes were examined as described previously [60, 61].

### RNA isolation

For isolation of RNA, 200 μl of plasma in 200 μl of PBS was thawed on ice and lysed with an equal volume of 2x Denaturing Solution (Life Technologies). To normalize sample-to-sample variation in plasma or exosomal RNA isolation, 25 fmol of synthetic *C. elegans* miR cel-miR-39 (QIAGEN, Valencia, CA) was added to each denatured sample [47]. Total RNA was extracted from 200 μl of plasma using miRNeasy Serum/Plasma Kits (QIAGEN) according to the manufacturer's instructions. RNA was isolated from blood cells and cultured cells by the Trizol (Life Technologies) method according to the manufacturer's protocol. The quality and quantity of the RNA was evaluated by ratios of 260/280 and 260/230 using NanoDrop spectrophotometry (NanoDrop, Wilmington, DE).

### TaqMan miR assay

Levels of miR-155 in cultured cells and blood cells were assessed by use of TaqMan miR Assays (Life Technologies), as described previously [62, 63]. Human miR-155 TaqMan primers and probes were purchased from Life Technologies. The average relative amounts were determined using the comparative method (2^-ΔCt^) against endogenous human *RNU6B* as a control.

### Nest-qPCR analysis

Due to the low amounts of miR-155 in plasma and exosomes, nest-qPCR analyses were performed to measure miR-155. Briefly, 5 μl of RNA in 20-μl reactions was reverse-transcribed using the miScript II RT Kit (QIAGEN) according to the manufacturer's protocol. cDNA (2 μl) was added to 20-μl reactions for pre-amplification PCR as described previously [47]. Then, 2 μl of the PCR products were used as templates for real-time PCR using a LightCycler 480 Real Time PCR System (Roche Applied Sciences, Indianapolis, IN) with miScript SYBR Green PCR kits (QIAGEN) at 95°C for 2 min, followed by 40 cycles of 95°C for 15 sec and 60°C for 1 min. The relative quantities of miR-155 in plasma were determined by use of an aCt value against the spiked-in control cel-miR-39 (QIAGEN), as described previously [49]. The relative quantities in exosomes were determined by the comparative method (2^-ΔCt^) with the spiked-in control cel-miR-39 (QIAGEN). The qPCR primer for human miR-155 is listed in Supplementary Table 1.

### Western blots

Western blotting was performed as previously described [36, 38, 58]. For nuclear proteins, the cells were first incubated in buffer A [10 mmol/L HEPES (pH 7.8), 10 mmol/L KCl, 2 mmol/L MgCl_2_, 0.1 mmol/L EDTA, 1% NP40, and protease inhibitors], and the pellet, obtained by centrifugation, was suspended in buffer B [50 mmol/L HEPES (pH 7.8), 300 mmol/L NaCl, 50 mmol/L KCl, 0.1 mmol/L EDTA, 10% (v/v) glycerol, and protease inhibitors].

### RNA interference (RNAi)-mediated gene knockdown

In 6-well plates, cells were cultured until 80% confluent in DMEM with 10% FBS with complete growth medium but without antibiotics. RNAi (20 pmol) and 10μl Lipofectamine 3000 were mixed in 250 μl of Opti-MEM I medium without serum and incubated for 20 minutes at room temperature. RNAi-Lipofectamine 3000 complexes (250 μl) were added into each well, giving a final RNAi concentration of 10 nM. After 8 hours, medium was changed to complete growth medium (DMEM with 10% FBS but without antibiotics). The cells were incubated for 48 hours at 37°C in a CO2 incubator until assayed for gene knockdown.

### Datasets, gene expression data analysis and annotation

TCGA Data Portal was used to download samples of invasive breast carcinomas (n=1,100), as described previously [64]. The RNAseqV2 level 3 data, which includes fragments per kilobase of exon per million fragments mapped-normalized gene level data, were used before statistical analysis. The idf and sdrf files were downloaded for sample mapping and annotation, and clinical outcomes data were downloaded for correlation analyses. Gene-level normalized expression data were used in Partek Genomic Suite (PGS, St. Louis, MO) for additional normalization, statistics, and annotation. False discovery rate (FDR) corrections (Benjamini-Hochberg methods) were applied for purpose of testing multiple hypotheses. In addition, the GEO datasets were downloaded from NCBI databases for comparison analyses between normal breast tissues and breast cancer tissues and between breast cancer subtypes.

### Statistical analyses

Continuous variables were summarized using sample size, mean, standard deviation (SD), median, minimum, and maximum values. For each group, the distribution of data was evaluated using a one-sample Kolmogorov-Smirnov test. In samples with normal distributions, the means of the variables were compared using a two-tailed *t* test between two groups. In samples with non-normal distributions, the medians of the variable between two groups were compared by a Mann-Whitney test. Analysis of variance (ANOVA), one- and two-way, were used to test for overall differences, followed by a protected least significant difference test for differences between groups. The Pearson correlation coefficient (*r*) was used to measure the strength of a linear association between expressions of *FOXP3* and *MIR155HG*. ROC curves were used to assess the diagnostic accuracy of plasma miR-155, and the sensitivity and specificity of the optimum cut-off point were defined as those values that maximized the AUC. All data were entered into an access database using Excel 2013 and analyzed with SPSS (version 20; IBM, Armonk, NY), StatView (version 5.0.1), and SAS Institute Inc., Cary, NC.

## SUPPLEMENTARY MATERIALS FIGURES AND TABLES


