# Supplementary material for: MicroRNA-155, induced by FOXP3 through transcriptional repression of BRCA1, is associated with tumor initiation in human breast cancer
Source: Oncotarget. 2017 May 11;8(25):41451–64. doi: 10.18632/oncotarget.17816 (PMC5522316; doi:10.18632/oncotarget.17816)
Supplement: Supplementary file 1 [file oncotarget-08-41451-s001.pdf]

MicroRNA-155, induced by FOXP3 though transcriptional repression of *BRCA1*, is associated with tumor initiation in human breast cancer

SUPPLEMENTARY MATERIALS

SUPPLEMENTARY FIGURES AND TABLES

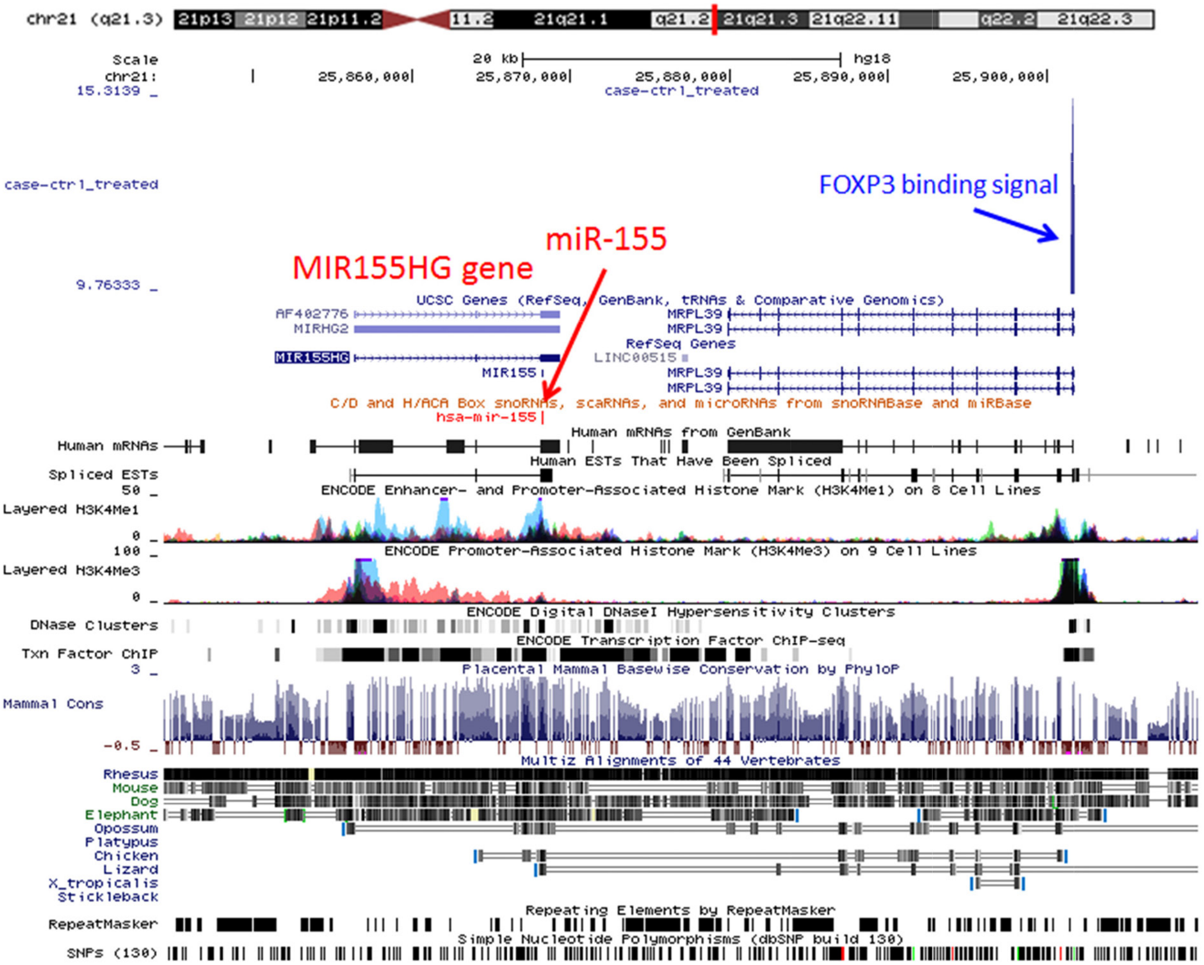

Supplementary Figure 1: No binding signals of FOXP3 in the promoter region of miR-155. The chromatin immunoprecipitation-sequencing (ChIP-seq) data as custom tracks within the UCSC genome browser were used to identify the FOXP3 binding sites by ChIP-seq peak detection. The blue arrow indicates the FOXP3 binding site, and the red arrow indicates the miR-155 locus. Chr, chromosome; kb, kilobase.

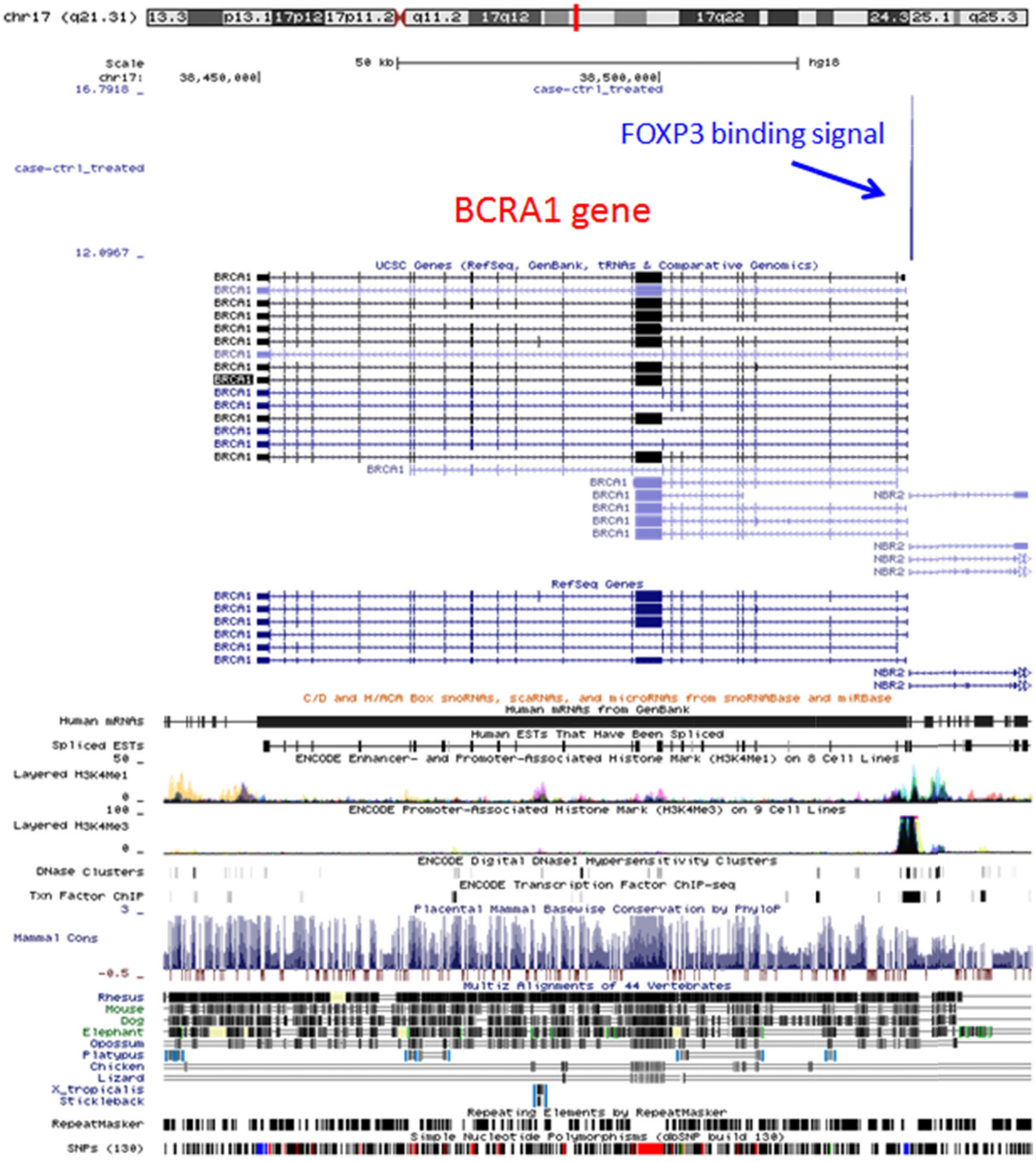

Supplementary Figure 2: Identification of a FOXP3 binding signal in the promoter region of *BRCA1*. The ChIP-seq data as custom tracks within the UCSC genome browser were used to identify the FOXP3 binding sites by ChIP-seq peak detection. The blue arrow indicates the FOXP3 binding site. Chr, chromosome; kb, kilobase.

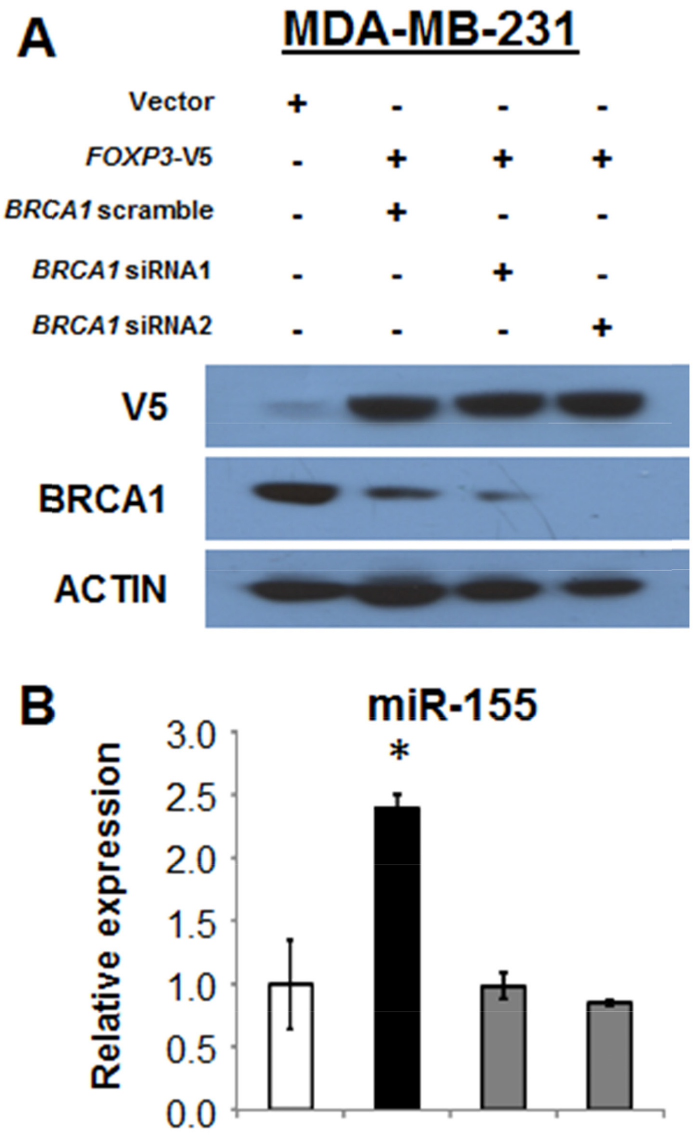

Supplementary Figure 3: FOXP3-BRCA1-miR-155 axis in MDA-MB-231 cells. (A) Representative Western blots showing FOXP3 and BRCA1 expression after transfection of the cells with empty vector or *FOXP3*-V5 and *BRCA1* siRNAs or a scramble control. **(B)** The expression of miR-155 in FOXP3-V5 transfected MDA-MB-231 cells before and after *BRCA1* silencing. *RNU6B* was used as an endogenous control. The expression of miR-155 in cells without transfections of *FOXP3*-V5 and *BRCA1* siRNAs as a reference, 1.0. * $p < 0.05$, one-way ANOVA followed by protected least significant difference test. All experiments were repeated three times.

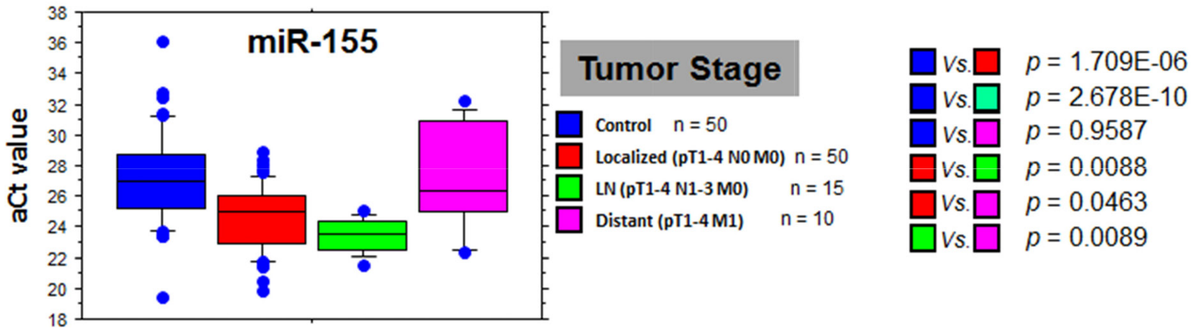

Supplementary Figure 4: Plasma levels of miR-155 in breast cancer patients with various tumor stages. Thebox-and-whisker plots of plasma miR-155 levels are presented as adjusted PCR cycle threshold (aCt) values for patients with tumor stages. The aCt value of each miR was adjusted by the spiked-in control cel-miR-39. For samples with normal distributions, the means of the variables were compared using a two-tailed *t* test between two groups. In samples with non-normal distributions, the medians of the variable between two groups were compared using a Mann-Whitney test. Local, localized breast cancer; LN, lymph node metastasis; Meta, distant metastasis. All experiments were repeated three times.

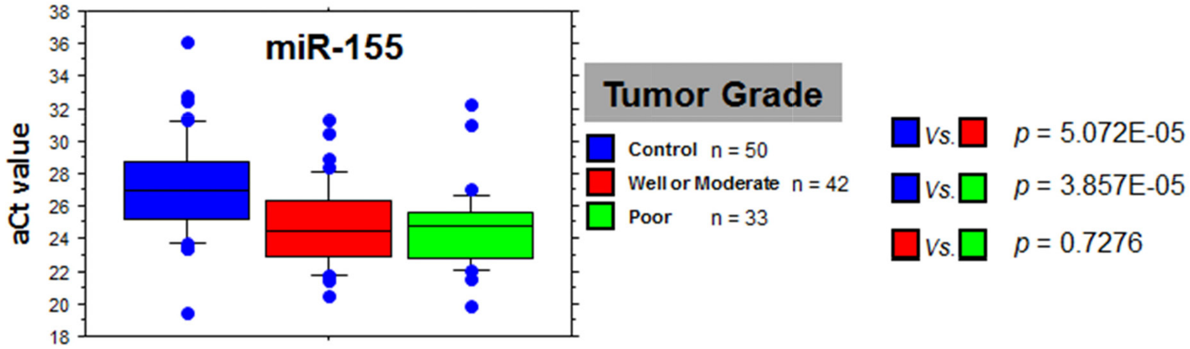

Supplementary Figure 5: Plasma levels of miR-155 in breast cancer patients with various tumor grades. The box-and-whisker plots of plasma miR-155 levels are presented as adjusted PCR cycle threshold (aCt) values for patients with tumor grades. The aCt value of each miR was adjusted by the spiked-in control cel-miR-39. In samples with normal distributions, the means of the variables were compared using a two-tailed *t* test between two groups. In samples with non-normal distributions, the medians of the variable between two groups were compared using a Mann-Whitney test. Well, well-differentiated tumor; Moderate, moderately differentiated tumor; Poor, poorly differentiated tumor. All experiments were repeated three times.

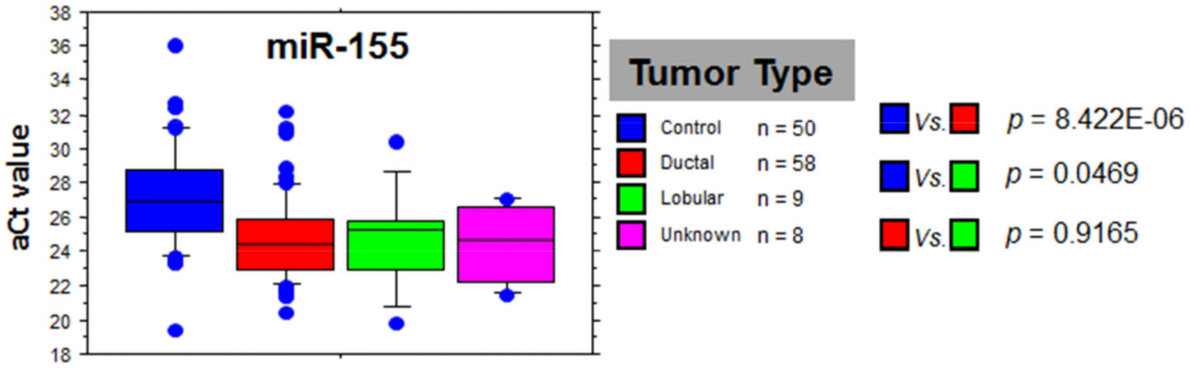

Supplementary Figure 6: Plasma levels of miR-155 in breast cancer patients with various tumor subtypes. The box-and-whisker plots of plasma miR-155 levels are presented as adjusted PCR cycle threshold (aCt) values for patients with tumor subtypes. The aCt value of each miR was adjusted by the spiked-in control cel-miR-39. In samples with normal distributions, the means of the variables were compared using a two-tailed *t* test between two groups. In samples with non-normal distributions, the medians of the variable between two groups were compared using a Mann-Whitney test. Ductal, invasive lobular carcinoma; Lobular, invasive ductal carcinoma; Unclassified, unclassified tumor type. All experiments were repeated three times.

Supplementary Table 1: Primer, siRNA and shRNA sequence used in this study

Primer Name	Sequence
Human BRCA1-realtime-F	TGGAGTTGATCAAGGAACCTG
Human BRCA1-realtime-R	GCCCTTTCTTCTGGTTGAGAA
Human TP63-realtime-F	TTGGGCTGTTCATCATGTCTG
Human TP63-realtime-R	TCCAGATCGCATGTCGAAAT
Human RAD51-realtime-F	TGTTGCCTATGCGCCAAA
Human RAD51-realtime-R	TTGGTGGAATTCAGTTGCAG
Human GAPDH-realtime-F	CCCCTTCATTGACCTCAACTACAT
Human GAPDH-realtime-R	CGCTCCTGGAAGATGGTGA
has-miR-155-5p-realtime-PCR	CCCTTAATGCTAATCGTGATAGGGGT
Human BRCA1 siRNA-1	CUAGAAAUCUGUUGCUAUG
Human BRCA1 siRNA-2	CAGCUACCCUUCCAUCAUA
Human FOXP3 shRNA-1	GCTTCATCTGTGGCATCATCC
Human FOXP3 shRNA-2	GAGTCTGCACAAGTGCTTTGT

Supplementary Table 2: The difference of methods, meaterials or patient population among the present and previous reports

Publications	Methods	Materials	Endogenous controls	Population
The present study	miScript miRNA PCR	Plasma	Cel-miR-39	American
TCGA dataset	RNA-seq	Tissues *	Self	American
Mol Cell Biol. 2008; 28: 6773-84.	mirVana qRT-PCR	Tissues	U6	American
J Surg Oncol. 2012; 106: 260-6.	Tagman miRNA assay	Tissues	U6	Chinese
Breast Cancer Res. 2010; 12: R90.	Tagman miRNA assay	Serum	miR-16	German
BMC Cancer. 2014; 14: 448.	Tagman miRNA assay	Serum	Let-7a	Czech
Clinical chemistry. 2013; 59: 1489-96.	Tagman miRNA assay	Serum	miR-16	German

* At least 80% of the cells in sample were required to be cancer cells.
